# 601. Characterizing Modifiable Risk Factors for Liposomal Amphotericin B (L-AMB) Nephrotoxicity

**DOI:** 10.1093/ofid/ofac492.653

**Published:** 2022-12-15

**Authors:** Kate Bialick, Teresa Leu, Catherine Liu, Frank P Tverdek

**Affiliations:** University of Washingotn, Seattle, Washington; University of Washington, Seattle, Washington; Fred Hutchinson Cancer Research Center, Seattle, Washington; Seattle Cancer Care Alliance, Seattle, Washington

## Abstract

**Background:**

Liposomal amphotericin B (L-AMB) is a broad spectrum anti-fungal utilized in the treatment of serious fungal infections. Despite its liposomal formulation aimed at reducing toxicity, it is still associated with relatively high rates of nephrotoxicity. Few studies have attempted to assess the effectiveness of mitigation strategies to lower the risk of L-AMB-associated nephrotoxicity.

**Methods:**

The purpose of this study is to characterize the time course of nephrotoxicity secondary to L-AMB, evaluate modifiable risk factors for L-AMB-related nephrotoxicity, and identify potential interventions to minimize nephrotoxicity. A retrospective chart review of inpatients who received L-AMB: Oct 1, 2020 through Jan 31, 2022 at the University of Washington Medical Center (UWMC) and Seattle Cancer Care Alliance (SCCA). The primary objective was to identify the incidence and onset of L-AMB related nephrotoxicity. Eligible patients ≥18 years old with a history of hematologic malignancy, solid organ transplant, or bone marrow transplant were included. Univariable associations were evaluated with either Chi-square or T-test.

**Results:**

A total of 132 patients were reviewed with 77 patient courses included. The baseline characteristics are depicted in TABLE 1. The mean dose of L-AMB was 4.9 mg/kg with an average treatment duration of 8.2 days. Nephrotoxicity occurred in 33.8 % of patients with median onset of 3 days. IV hydration occurred in 88.5 and 68% of patients pre and post, respectively. Patients had at least one concomitant nephrotoxin (87 %) with an average of 2. Comparing those patients with nephrotoxicity and those without, age and L-AMB duration, were associated with nephrotoxicity.

**Table 1:**
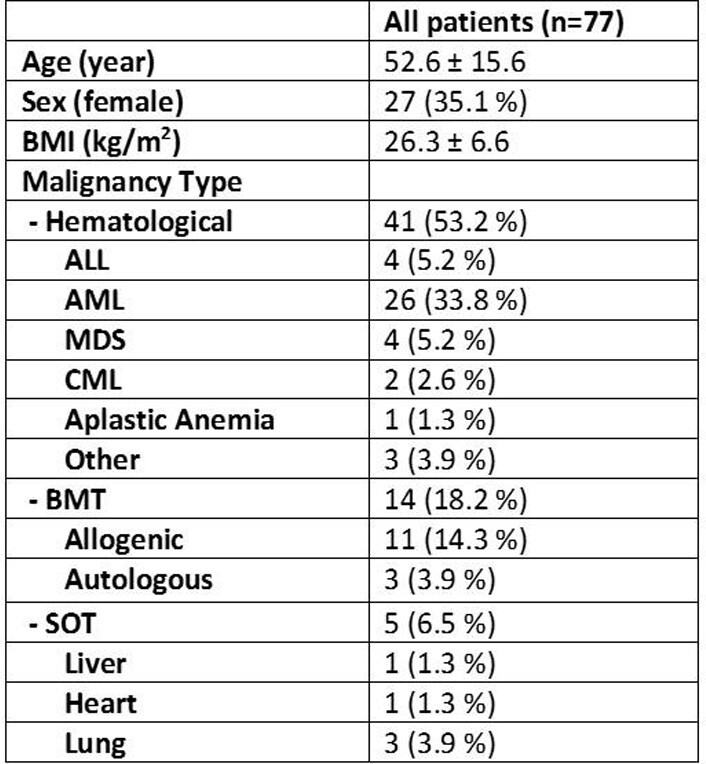
- Baseline Characteristics

**Table 2:**
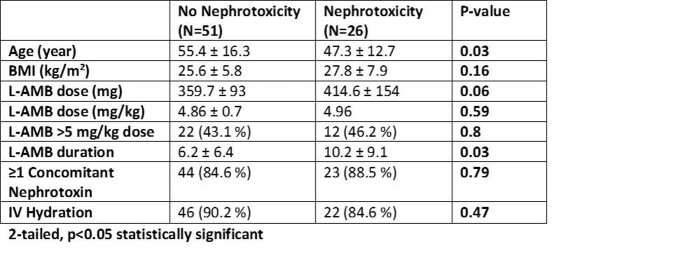
- Nephrotoxicity Risk Factors

**Conclusion:**

L-AMB nephrotoxicity occurred at a relatively high rate in this patient population despite high utilization of toxicity mitigating strategies such as IV hydration. Nephrotoxicity was associated with longer durations of therapy. Further investigation is necessary to determine alternative strategies for nephrotoxicity prevention.

**Disclosures:**

**All Authors**: No reported disclosures.

